# Supervision Strategy Analysis on Price Discrimination of E-Commerce Company in the Context of Big Data Based on Four-Party Evolutionary Game

**DOI:** 10.1155/2022/2900286

**Published:** 2022-03-18

**Authors:** Meng Xiao

**Affiliations:** School of Management, Shenyang University of Technology, Shenyang 110870, China

## Abstract

This paper focuses on the phenomenon of “big data killing” implied in e-commerce and discusses how to take the government as the lead to coordinately supervise the price discrimination behavior of e-commerce companies towards loyal customers. First, the four-party evolutionary game model of the government regulatory department, e-commerce platform, e-commerce company, and consumer is built. Second, the stability of the strategy choice of each game subject is analyzed. On this basis, the evolutionary stable strategy in the system based on First Law of Lyapunov is explored. Finally, the influences of key elements on system evolution are simulated and analyzed by MATLAB2021. Results demonstrate that (1) the government supervision mechanism can effectively supervise the price discrimination of e-commerce company based on big data to loyal customers; (2) when the government chooses the strict supervision strategy, reducing the information supervision cost of the e-commerce platform and the strict supervision cost of the government enable the government and the e-commerce platform to coordinate supervision and make the e-commerce company incline to choose the nondifferential pricing strategy; (3) when the government chooses the loose supervision strategy, reducing the information supervision cost of the e-commerce platform and increasing the probability of consumer discovering differential pricing and the penalties for differential pricing of e-commerce company enable the e-commerce platform and consumer to coordinate supervision, and make the e-commerce company incline to choose the nondifferential pricing strategy. The results of this study can provide theoretical guidance for the government and companies to make beneficial strategic decisions in the development of e-commerce.

## 1. Introduction

With the rise of big data, e-commerce is becoming more and more prosperous. E-commerce can bring convenience to consumers with a variety of options and also collect consumer consumption data and draw user portraits by using big data technology [1]. While the application of algorithms injects new growth drivers into social and economic development, problems caused by the unreasonable application of algorithms such as algorithm discrimination, “big data killing,” and inducing addiction also profoundly affect the normal communication in the market and destroy the market order. Online supply chain stores have different pricing based on user location. On some online booking websites, the price of hotel rooms for Apple customer is higher than that for Windows customer. The well-known e-commerce company, Amazon, was found to use big data to “kill regular” [2]. It priced for different consumers according to their information and purchasing data on the platform. Loyal customers made purchase transactions based on their trust and path dependence on the Amazon platform, but due to the asymmetry of information in the transaction process, some regulars pay higher prices than strangers. This “big data killing” behavior has exposed the hidden dangers of moral hazard in the e-commerce market and makes the industry encounter an unprecedented crisis of trust. “Big data killing” has become an urgent problem to be solved in the fast development of online business [3].

The essence of big data killing is price discrimination. Price discrimination refers to formulating different price strategies for different customer groups. However, in traditional business, both “stranger” and “regular” may be discriminated against, while with the participation of algorithm technology, there are more “killing regular” in Internet business. Even in the process of “killing regular,” big data has become a necessary tool. Each platform will collect a lot of user information, and then the company uses technology to offer different prices and discounts for different customers based on the information. Traditionally, companies have not been able to predict the upper limit of the price that buyers want to pay, but based on the technology of big data, the companies can determine the maximum willing price with a high degree of accuracy with sophisticated algorithms [4]. As the collection of consumer data becomes more common, online companies are now more capable of price discrimination than ever [5]. As customer of the Internet commercial company, VIP customer with higher loyalty and stronger consumption power pay much more for the same service than new customers, but gain even lower service quality. Big data killing will cause a variety of harm. Moriarty [6] proposed that customer information is widely used in online retail pricing, and although the benefit of online retailers will increase, price discrimination can cause serious fairness concerns and even violation of regulations and laws. Antimonopoly issues in the digital economy, especially the antimonopoly issues of big data and discrimination algorithms, have been brought to the attention of experts and practitioners. “Killing regular” is algorithmic price discrimination, with which online platforms charge long-term customers higher prices. It is believed that this kind of price discrimination violates the law on antimonopoly and should be held accountable according to the relevant law. The Cyberspace Administration of China (CAC) issued the regulations on The Management of Algorithm Recommendation for Internet Information Services to regulate the “big data killing,” stepping into the era of strict supervision of the industry related to algorithm recommendation. The EU also prohibits discrimination on certain grounds and strictly regulates unfair business practices in B2C relationships [7].

Although some studies have carried out related discussion on the problem of big data killing [2, 8], some solutions are proposed [9, 10]. However, existing studies are mostly limited to the pricing between e-commerce companies and consumers [11, 12], the strategic choice between e-commerce platform and consumer, and the supervision strategy choice between e-commerce platform and government [9, 13]. There are few systematic studies on the four-party strategy composed of multiple subjects related to “big data killing.” Therefore, this study establishes an evolutionary game model dominated by government supervision that affects the decision-making of consumer, e-commerce company, and platform, analyzes and simulates that different supervision costs of government and e-commerce platform, consumer discovery levels, and the penalties for differential pricing of e-commerce company affect system equilibrium, evolutionarily stable strategy, and the pricing strategy of e-commerce company, and also establishes the platform-consumer-government collaborative supervision mechanism for e-commerce company pricing behavior. This research contributes to curbing the “big data killing” behavior of e-commerce company, enhance consumers' confidence in online shopping, and has a positive effect in promoting the development of e-commerce.

## 2. Related Literature

Existing research on price discrimination in e-commerce companies mainly focuses on three aspects: the prevalence of price discrimination by using customer information, the influence factors of price discrimination, and the supervision and management of price discrimination:

On the prevalence of price discrimination by e-commerce companies using customer information, although many media outlets provide various evidence of price discrimination, most of them are not based on scientific and systematic methods. Therefore, scholars have researched whether e-commerce companies use big data to discriminate against consumers in price. Botta and Klaus [14] qualitatively proposed that algorithmic price discrimination is different from offline differential pricing and is related to the collection of consumer information, which is a unique feature of the digital economy. With the wide application of big data and the gradual deepening of algorithm technology, the e-commerce company can price discriminate against consumers with great precision [4], and these were confirmed empirically [4, 15]. The pricing ecosystem of the online platform is a dynamic pricing system [15]. Algorithmic price discrimination [16], artificial intelligence techniques, and digital system fingerprints [15] enable the e-commerce company to have the ability of price discrimination. Price discrimination is not only widespread in the field of commodity sales, and there are also discrimination and price difference by using customer information in the field of online car-hailing [8] and the field of advertising recommendation [7, 17]. While consumers benefit from accurate recommendations, sellers may use this information to discriminate on price. Thus, price discrimination is not favored by people [18].

Scholars have done a lot of studies on the influence factors of price discrimination in e-commerce companies. Some scholars believe that the premise of “big data killing” is the information asymmetry between e-commerce company and customers [1]. Consumer information data is an influencing factor for the e-commerce company to be able to discriminate in price [19], such as consumer characteristics, location [14], etc., and these data also relate to consumers' privacy [12]. Nuccio and Marco [20] studied how pricing technology and information transparency are changing merchants' pricing behavior in online transactions. The price sensitivity and heterogeneity of consumers are factors that affect e-commerce company to set price differentials [11]. Some scholars have analyzed the effects of reference price and search cost on differential pricing and find that consumers' search cost has become one of the obstacles affecting consumers' online shopping, which has formed an unequal situation for consumers [21] and has become a tool for e-commerce companies to formulate differential prices [22, 23]. The target of “big data killing” of e-commerce companies is focused on loyal consumers, which has been confirmed by many scholars. For example, Tang et al. [24] found in the research on the group-buying market that with the improvement of consumer retention rate, the best strategy of sellers is changed from quality difference to price discrimination. Chandra and Lederman [25] argued that if consumers have differences in potential willingness to pay and brand loyalty, e-commerce companies may increase price differences among some consumers while reducing price differences among the other consumers. Although differential pricing is an important way for e-commerce companies to obtain profits [26], its focus on loyal consumers is contrary to the principle of fair pricing [24], which will reduce consumer satisfaction and create distrust [27, 28].

After the problem of “big data killing” was exposed, it has been attracted widespread attention by scholars, and its supervision and governance have also become an important research topic. Bar-Gill [29] proposed that the normative evaluation of price discrimination depends on the object of discrimination, and the algorithmic price discrimination has the advantages to improve efficiency, but it will harm consumers, which should be governed by rules set by regulators to seriously exploit the potential of personalization. Yu and Li [9] also believed that consumers' discovery and reporting of being “killed” is the mean to monitor price discrimination of e-commerce company. Xing et al. [3] found that when regulars account for a high proportion of platform customer, giving consumers the right to data portability can curb the phenomenon of “big data killing” to a certain extent. In addition to consumers' self-discovery of price discrimination by the e-commerce company, many scholars believe that with the help of government supervision [13], increasing penalties and the commission coefficient of government departments [30] can effectively reduce the “killing regular” pricing tendency of e-commerce platforms. However, in the supervision process of existing research, there was little distinction between e-commerce platform and company, and the research is carried out in a mixed way. Most of the discussions focus on the pricing of e-commerce platforms known for its scale. Differential pricing of e-commerce company on the platform is rarely discussed separately, and there is still a lack of research considering multichannel collaborative supervision.

Existing studies have adopted a variety of methods for the problem of price discrimination in the e-commerce company. For example, the dynamic pricing method is used in specific pricing. Lindgren et al. [31] studied dynamic pricing by intertemporal price discrimination theory and proposed that retailers should change prices randomly over time. Chevalier and Kashyap [32] proposed the method for aggregating prices when retailers use periodic sales to discriminate price against heterogeneous customers. Tremblay [5] designed more efficient Pareto price discrimination. Game methods are often used in the selection of pricing strategies. Choe et al. [33] analyzed pricing strategy with a two-stage dynamic game model. Zhou et al. [34] adopted two-stage game analysis on joint pricing and bandwidth demand optimization. On the game of price discrimination, the bounded rationality assumption in the evolutionary game makes the research more realistic [30], so many scholars use evolutionary game methods to study this problem [1, 13, 30, 35] and extended to multiple fields of online transactions, such as manufacturing business [36]. However, most studies are limited in the two-party game [22, 37], it is still unclear to analyze the relationship and role of e-commerce company, consumer, e-commerce platform, and the government in the “big data killing” problem system, and their decision-making mechanism needs further research.

Therefore, as the price discrimination of e-commerce companies is generated with new technologies, the existing research on this phenomenon is still in the exploratory stage. Most perspectives of the previous research are from both sides of the transaction in traditional business, and there are few differences in the analysis of the e-commerce platform and the companies in the platform. Moreover, the supervision on the differential pricing of the e-commerce company using big data technology to the loyal customers is not very perfect, and some policies and supervision methods are still under discussion. This study systematically analyzes the government, e-commerce platform, e-commerce company, and consumer involved in the supervision of “big data killing,” which makes up for the insufficiency of the existing research and provides useful help for further regulating such behavior.

## 3. Materials and Methods

### 3.1. Problem Description

The e-commerce company will use the platform to collect consumer information during the operation in the network platform. Based on the information provided by the platform, e-commerce company analyzes consumers and raise prices by judging their consumption habits. The pricing strategy of “big data killing” is price discrimination caused by e-commerce company using the feature of opaque information in the online transaction process to different pricing of consumers through big data and complex algorithms. This kind of behavior will bring consumers' distrust of e-commerce companies and e-commerce platforms, which is not conducive to the development of e-commerce. Therefore, both the government regulatory department and e-commerce platforms should take necessary measures to supervise the price discrimination behavior of e-commerce companies. This study mainly discusses the following three questions: (1) in the context of big data development, how can the government regulatory department take supervision measures to reduce the proportion of price discrimination by e-commerce company? (2) How can e-commerce platform be motivated to supervise information on e-commerce companies? (3) How can consumers be guided to actively safeguard their rights and interests and maintain consumption fairness.

**Figure 1:**
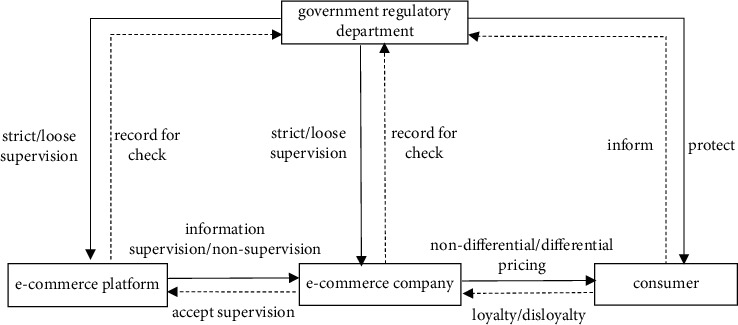
Game model logic relationship of multisubject supervision on e-commerce company pricing.

This study builds a multi-agent game model for the supervision of price discrimination in e-commerce companies involving the e-commerce platform, the e-commerce company, the consumer, and the government regulatory department. The logical relationship among four-party game subjects is shown in Figure 1.

### 3.2. Model Assumption

To build the multisubject supervision model of the e-commerce company pricing in the background of big data, the behavioral strategies of government regulatory department, e-commerce platform, e-commerce company, and consumer are studied, and the following assumptions are made.

**Assumption 1 .**

Government regulatory department, e-commerce platform, the e-commerce company, and consumer are selected as the game subjects. Each game subject is bounded rationality and pursues the maximization of their interests in e-commerce transactions. Due to the information asymmetry between game subjects, random behavior strategies, and interactive effects, the optimal strategy cannot be obtained through one game. It is necessary to continuously try and learn in multiple rounds of games to improve the strategy, to formulate the best match of behavioral decision. Therefore, the evolutionary game should be used to analyze the four-party equilibrium strategy. The proportion of e-commerce company implementing nondifferential pricing is represented as *x* (0 ≤ *x* ≤ 1), and the proportion of e-commerce company implementing differential pricing is denoted as (1 − *x*); the proportion of consumer loyalty is represented as *y* (0 ≤ *y* ≤ 1) and the proportion of consumer disloyalty is represented as (1 − *y*); the proportion of e-commerce platform to supervise company information is represented as *z* (0 ≤ *z* ≤ 1), and the proportion of e-commerce platform with information nonsupervision is denoted as (1 − *z*); the proportion of the government regulatory department strictly supervising e-commerce platform and company is denoted as *r* (0 ≤ *r* ≤ 1), and the proportion of loosely supervises e-commerce platform and the company is denoted as (1 − *r*).

**Assumption 2 .**

The benefit of nondifferential pricing of the e-commerce company is *P*_*n*_, and the basic benefit of differential pricing is *P*_*d*_. When the e-commerce company implements differential pricing for loyal consumer, additional benefit *∆P* can be obtained due to the increase in selling price, and *P*_*d*_ *<* *P*_*n*_ *<* *P*_*d*_*+∆P*. The probability of loyal consumers discovering differential pricing of the e-commerce company is *α*. When consumer purchases goods, the utility obtained by the loyal consumer is *U*_*l*_, and the utility obtained by the disloyal consumer is *U*_*d*_, and *U*_*l*_ *>* *U*_*d*_. The reputation value of the loyal consumer to the e-commerce company is *T*_*e*_ and the reputation value of the loyal consumer to the e-commerce platform is *T*_*p*_.

**Assumption 3 .**

When the government strictly supervises, if price discrimination of the e-commerce company is found, loyal consumers who are subject to differential pricing will be compensated with the compensation amount of *M*; When the government loosely supervises, if the loyal consumer is the price-sensitive consumer, he may use Internet information for comparison and analysis, and then find that he has been “killed”. If the cost of reporting is small and the procedure is simple, the consumer will carry out to inform the government regulatory department, and then the e-commerce company must be forced to compensate the consumer. The consumer's complaint cost is *C*_*c*_.

**Assumption 4 .**

The normal benefit that the government obtains from the operation of the e-commerce platform is *S*. The cost of strict supervision by government departments is *C*_*g*_. The social benefit obtained by the government is *R* if there is no price discrimination by the e-commerce company. If the government adopts the loose supervision policy, consumer complaints will bring social reputation loss as *N*. After receiving the information, the e-commerce company for price discrimination will be penalized by the government regulatory department, and the fine will be *I*_*e*_.

**Assumption 5 .**

The price discrimination of e-commerce company depends on the information provided by the platform. The benefit of the platform reasonably providing information to the e-commerce company is *W*, and the cost of the platform information supervision on e-commerce company is *C*_*p*_. When the e-commerce platform finds the price discrimination of e-commerce company on the consumer, the fine to e-commerce company is *F*. The e-commerce platform and consumers share this fine in the ratio of *β* and 1 − *β*. When the government finds price discrimination by the e-commerce company, it will impose the fine of *I*_*p*_ for the platform's unfavorable supervision to e-commerce company information.

The parameters are described in Table 1.

### 3.3. Model Framework

According to the above analysis, the mixed-strategy game matrix of the four-party game subjects of government regulatory department, e-commerce platform, e-commerce company, and consumer is shown in Table 2.

### 3.4. Model Analysis

#### 3.4.1. Strategy Stability Analysis of the E-Commerce Company

Assuming that the expected benefit of the e-commerce company when choosing the nondifferential pricing strategy is *U*_*11*_, the expected benefit of the e-commerce company when choosing the differential pricing strategy is *U*_*12*_, and the average expected benefit of the e-commerce company is $\bar{U_{1}}$, which are defined as follows:

(1) $$\begin{array}{c} U_{11}=yzr\left(P_{n}+T_{e}\right)+\left(1-y\right)zrP_{n}+y\left(1-z\right)r\left(P_{n}+T_{e}\right)+\left(1-y\right)\left(1-z\right)rP_{n}\\ +yz\left(1-r\right)\left(P_{n}+T_{e}\right)+\left(1-y\right)z\left(1-r\right)P_{n}+y\left(1-z\right)\left(1-r\right)\left(P_{n}+T_{e}\right)+\left(1-y\right)\left(1-z\right)\left(1-r\right)P_{n}\\ =P_{n}+yT_{e},\\ U_{12}=yzr\left(P_{d}+\Delta P+T_{e}-M-I_{e}-F\right)+\left(1-y\right)zr\left(P_{d}-I_{e}-F\right)\\ +y\left(1-z\right)r\left(P_{d}+\Delta P+T_{e}-M-I_{e}\right)+\left(1-y\right)\left(1-z\right)r\left(P_{d}-I_{e}\right)\\ +yz\left(1-r\right)\left(P_{d}+\Delta P+T_{e}-\alpha M-\alpha I_{e}-F\right)+\left(1-y\right)z\left(1-r\right)\left(P_{d}-F\right)\\ +y\left(1-z\right)\left(1-r\right)\left(P_{d}+\Delta P+T_{e}-\alpha M-\alpha I_{e}\right)+\left(1-y\right)\left(1-z\right)\left(1-r\right)P_{d}\\ =P_{d}+y\left(\Delta P+T_{e}\right)-y\left(M+I_{e}\right)\left[r+\left(1-r\right)\alpha \right]-\left(1-y\right)rI_{e}-zF,\\ \bar{U_{1}}=xU_{11}+\left(1-x\right)U_{12}. \end{array}$$

According to the Malthusian dynamic equation, the replication dynamic equation of the e-commerce company is obtained as follows:

(2) $$\begin{array}{c} F\left(x\right)=\frac{\text{d}x}{\text{d}t}\\ =x\left(U_{11}-\bar{U_{1}}\right)\\ =x\left(1-x\right)\left\{P_{n}-P_{d}-y\Delta P+y\left(M+I_{e}\right)\left[r+\left(1-r\right)\alpha \right]+\left(1-y\right)rI_{e}+zF\right\}. \end{array}$$

The first partial derivative of *F* (*x*) for *x* is as follows:

(3) $$\begin{array}{c} F_{x}^{\prime }\left(x\right)=\left(1-2x\right)\left\{P_{n}-P_{d}-y\Delta P+y\left(M+I_{e}\right)\left[r+\left(1-r\right)\alpha \right]+\left(1-y\right)rI_{e}+zF\right\}. \end{array}$$

Based on the stability theorem of differential equations, the e-commerce company implements the strategy of nondifferential pricing in the stable state must meet the conditions: *F*(*x*) = 0, and *F*_*x*_′(x) < 0.

**Proposition 1 .**

When *r* *>* *r*_*0*_, the stable strategy of the e-commerce company is nondifferential pricing; when *r* *<* *r*_*0*_, the stable strategy of the e-commerce company is differential pricing; when *r* *=* *r*_*0*_, the e-commerce company cannot determine the stable strategy. Where the threshold is as follows:

(4) $$\begin{array}{c} r_{0}=\frac{P_{d}+y\Delta P-P_{n}-\alpha y\left(M+I_{e}\right)-zF}{\left(1-\alpha \right)yM+\left(1-\alpha y\right)I_{e}}. \end{array}$$

**Proof**

Assume *H*(*r*)=*P*_*n*_ − *P*_*d*_ − *y*Δ*P*+*y*(*M*+*I*_*e*_)[*r*+(1 − *r*)*α*]+(1 − *y*)*rI*_*e*_+*zF*, when *y*[*M* − *α*(*M*+*I*_*e*_)] > 0, (∂*H*/∂*r*) > 0, then *H (r)* is considered to be an increasing function of *r*. When *r* *>* *r*_*0*_, *H (r)* > 0, *F*(*x*)*|*_*x*=1_=0, and *F*_*x*_′(*x*)*|*_*x*=1_ < 0, so *x* = 1 has stability; When *r* *<* *r*_*0*_, *H (r)* < 0, *F*(*x*)*|*_*x*=0_=0, and *F*_*x*_′(*x*)*|*_*x*=0_ < 0, so *x* = 0 has stability; when *r* *=* *r*_*0*_, *H (r)* = 0, *F*(*x*)=0, and *F*_*x*_′(*x*)=0, so *x* is stable at all levels in the range of 0 to 1, that is, the company's strategy does not change over time, regardless of the proportion of company choosing to price differentially.

Proposition 1 states that the increase of the proportion of the government strict supervision to e-commerce company will change the stable strategy of e-commerce company from differential pricing to nondifferential pricing; Similarly, the decline of the proportion of the government strict supervision to e-commerce company will change the stable strategy of e-commerce company from nondifferential pricing to differential pricing. Therefore, the government's strict supervision for e-commerce company is essential, and the government should take measures to improve strict supervision for the e-commerce company.

**Figure 2:**
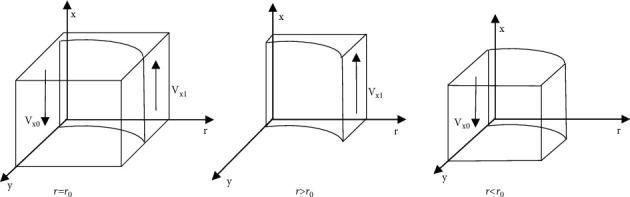
Phase diagram of strategy evolution of e-commerce company.

Based on Proposition 1, the phase diagram of the strategy evolution of e-commerce company is shown in Figure 2.

Inference 1: with the increase of the value of *P*_*n*_, *M*, *I*_*e*_, *F*, and *α*, the e-commerce company is more inclined to implement the nondifferential pricing strategy, when other parameters remain unchanged. Similarly, with the increase of the value of *P*_*d*_ and *∆P*, the e-commerce company is more inclined to implement the differential pricing strategy. It shows that the proportion of e-commerce company implementing nondifferential pricing strategy is directly proportional to the benefits of nondifferential pricing, the fines imposed by the government and platform on e-commerce company for differential pricing and the probability of consumers' discovery, and inversely proportional to the benefits of e-commerce company implementing differential pricing strategy.

**Proof**

Since *r*_0_=(*P*_*d*_+*y*Δ*P* − *P*_*n*_ − *αy*(*M*+*I*_*e*_) − *zF*/(1 − *α*)*yM*+(1 − *αy*)*I*_*e*_), the volume of *V*_*x1*_ in Figure 2 represents the proportion of nondifferential pricing by the e-commerce company, and the corresponding volume of *V*_*x0*_ represents the proportion of differential pricing by the e-commerce company. When the value of *P*_*n*_, *M*, *I*_*e*_, *F*, and *α* gradually increases, the value of *r*_*0*_ will gradually decrease, and the volume of *V*_*x1*_ will increase at this time, indicating that the proportion of e-commerce company to implement nondifferential pricing increases; When the value of *P*_*d*_ and *∆P* gradually increases, the value of *r*_*0*_ will gradually increase, and the volume of *V*_*x1*_ will decrease at this time, indicating that the proportion of e-commerce company to implement nondifferential pricing decreases.

#### 3.4.2. Strategy Stability Analysis of the Consumer

Assuming that the expected benefit of the consumer when choosing loyalty strategy to e-commerce company is *U*_*21*_, the expected benefit of the consumer when choosing disloyalty strategy to e-commerce company is *U*_*22*_, and the average expected benefit of the consumer is $\bar{U_{2}}$, which are defined as follows:

(5) $$\begin{array}{c} U_{21}=xzrU_{l}+\left(1-x\right)zr\left[U_{l}-\Delta P+M+\left(1-\beta \right)F\right]\\ +x\left(1-z\right)rU_{l}+\left(1-x\right)\left(1-z\right)r\left(U_{l}-\Delta P+M\right)\\ +xz\left(1-r\right)U_{l}+\left(1-x\right)z\left(1-r\right)\left[U_{l}-\Delta P-C_{c}+\alpha M+\left(1-\beta \right)F\right]\\ +x\left(1-z\right)\left(1-r\right)U_{l}+\left(1-x\right)\left(1-z\right)\left(1-r\right)\left(U_{l}-\Delta P-C_{c}+\alpha M\right)\\ =U_{l}-\left(1-x\right)\Delta P+\left(1-x\right)\left[M+\left(1-r\right)\alpha M\right]\\ -\left(1-x\right)\left(1-r\right)C_{c}\\ +\left(1-x\right)z\left(1-\beta \right)F,\\ U_{22}=xzrU_{d}+\left(1-x\right)zrU_{d}\\ +x\left(1-z\right)rU_{d}\\ +\left(1-x\right)\left(1-z\right)rU_{d}\\ +xz\left(1-r\right)U_{d}\\ +\left(1-x\right)z\left(1-r\right)U_{d}\\ +x\left(1-z\right)\left(1-r\right)U_{d}\\ +\left(1-x\right)\left(1-z\right)\left(1-r\right)U_{d}\\ =U_{d},\\ \bar{U_{2}}=yU_{21}+\left(1-y\right)U_{22}. \end{array}$$

According to the Malthusian dynamic equation, the replication dynamic equation of consumer is obtained as follows:

(6) $$\begin{array}{c} F\left(y\right)=\frac{\text{d}y}{\text{d}t}\\ =y\left(U_{21}-\bar{U_{2}}\right)\\ =y\left(1-y\right)\left\{\begin{array}{c} U_{l}-U_{d}-\left(1-x\right)\Delta P\\ +\left(1-x\right)\left[M+\left(1-r\right)\alpha M\right]\\ -\left(1-x\right)\left(1-r\right)C_{c}+\left(1-x\right)z\left(1-\beta \right)F \end{array}\right\}. \end{array}$$

The first partial derivative of *F* (*y*) for *y* is as follows:

(7) $$\begin{array}{c} F_{y}^{\prime }\left(y\right)=\left(1-2y\right)\left\{U_{l}-U_{d}-\left(1-x\right)\Delta P+\left(1-x\right)\left[M+\left(1-r\right)\alpha M\right]-\left(1-x\right)\left(1-r\right)C_{c}+\left(1-x\right)z\left(1-\beta \right)F\right\}. \end{array}$$

Based on the stability theorem of differential equations, consumer implements the strategy of loyalty in the stable state must meet the conditions: *F*(*y*) = 0, and *F*_*y*_′(y) < 0.

**Proposition 2 .**

When *x* *>* *x*_*0*_, the stable strategy of the consumer is loyalty; when *x* *<* *x*_*0*_, the stable strategy of the consumer is disloyalty; when *x* *=* *x*_*0*_, the consumer cannot determine the stable strategy. Where the threshold is as follows:

(8) $$\begin{array}{c} x_{0}=\frac{U_{l}-U_{d}+M-\Delta P+\left(1-r\right)\left(\alpha M-C_{c}\right)+\text{z}\left(1-\beta \right)\text{F}}{M-\Delta P+\left(1-r\right)\left(\alpha M-C_{c}\right)+\text{z}\left(1-\beta \right)\text{F}}. \end{array}$$

**Proof**

Assume *H*(*x*)=*U*_*l*_ − *U*_*d*_ − (1 − *x*)Δ*P*+(1 − *x*)[*M*+(1 − *r*)*αM*] − (1 − *x*)(1 − *r*)*C*_*c*_+(1 − *x*)*z*(1 − *β*)*F*, when *M* − Δ*P*+(1 − *r*)(*αM* − *C*_*c*_)+*z*(1 − *β*)*F* > 0, (∂*H*/∂*x*) > 0, *H (x)* is considered to be an increasing function of *x*. When *x* *>* *x*_*0*_, *H (x)* *>* 0, *F*(*y*)*|*_*y*=1_=0, and *F*_*y*_′(*y*)*|*_*y*=1_ < 0, so *y* = 1 has stability; When *x* *<* *x*_*0*_, *H (x)* < 0, *F*(*y*)*|*_*y*=0_=0, and *F*_*y*_′(*y*)*|*_*y*=0_ < 0, so *y* = 0 has stability; When *x* *=* *x*_*0*_, *H (x)* = 0, *F*(*y*)=0, and *F*_*y*_′(*y*)=0, so *y* is stable at all levels in the range of 0 to 1, that is, the consumer's strategy does not change over time, regardless of the proportion of consumer choosing to be loyal.

Proposition 2 states that the increase of the proportion of nondifferential pricing of e-commerce company will change the stable strategy of consumer from disloyalty to loyalty; Similarly, the decline of the proportion of nondifferential pricing of e-commerce company will change the stable strategy of consumer from loyalty to disloyalty. Therefore, e-commerce company should reduce the degree of difference in pricing for consumers and try to retain consumers.

**Figure 3:**
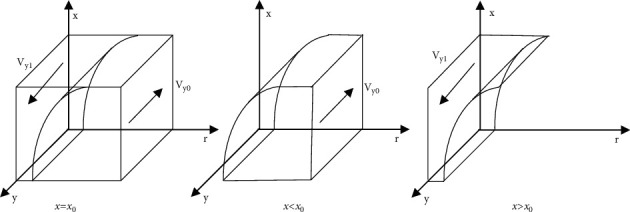
Phase diagram of strategy evolution of consumer.

Based on Proposition 2, the phase diagram of the strategy evolution of consumer is shown in Figure 3.

Inference 2: with the increase of the value of *U*_*l*_, *M*, *F*, *α*, and *β*, the consumer is more inclined to be loyalty strategy to the e-commerce company, when other parameters remain unchanged. Similarly, with the increase of the value of *U*_*d*_, *∆P*, and *C*_*c*_, the consumer is more inclined to be disloyalty strategy to the e-commerce company. It shows that the proportion of consumer being loyalty strategy to e-commerce company is directly proportional to the utility obtained by the loyal consumer from purchasing goods, the fines imposed by the government and e-commerce platform for differential pricing of e-commerce company, and the probability of consumers' discovery, and inversely proportional to the utility obtained by the disloyal consumer in purchasing goods, the additional benefit obtained by the e-commerce company in implementing differential pricing, the proportion of fines imposed by the platform to the e-commerce company and the cost of consumer complaints.

**Proof**

Since *x*_0_=1 − (*U*_*l*_ − *U*_*d*_/Δ*P*+(1 − *r*)*C*_*c*_ − [1+(1 − *r*)*α*]*M* − z(1 − *β*)*F*), the volume of *V*_*y1*_ in Figure 3 represents the proportion of loyalty to e-commerce company by the consumer, and the corresponding volume of *V*_*y0*_ represents the proportion of disloyalty to e-commerce company by the consumer. When the value of *U*_*l*_, *M*, *I*_*e*_, *F*, and *α* gradually increases, the value of *x*_*0*_ will gradually decrease, and the volume of *V*_*y1*_ will increase at this time, indicating that the proportion of loyalty to e-commerce company by the consumer increases; When the value of *U*_*d*_, *∆P*, *β* and *C*_*c*_ gradually increase, the value of *x*_*0*_ will gradually increase, and the volume of *V*_*y1*_ will decrease at this time, indicating that the proportion of loyalty to e-commerce company by consumer decreases.

#### 3.4.3. Strategy Stability Analysis of E-Commerce Platform

Assuming that the expected benefit of the e-commerce platform when choosing the information supervision strategy is *U*_*31*_, the expected benefit of the e-commerce platform when choosing the information nonsupervision strategy is *U*_*32*_, and the average expected benefit of the e-commerce platform is $\bar{U_{3}}$, which are defined as follows:

(9) $$\begin{array}{c} U_{31}=xyr\left(W-C_{p}+T_{P}\right)+x\left(1-y\right)r\left(W-C_{p}\right)\\ +\left(1-x\right)\left(yrW-C_{p}+T_{P}+\beta F\right)\\ +\left(1-x\right)\left(1-y\right)r\left(W-C_{p}+T_{P}\right)\\ +xy\left(1-r\right)\left(W-C_{p}+\beta F\right)\\ +x\left(1-y\right)\left(1-r\right)\left(W-C_{p}\right)\\ +\left(1-x\right)y\left(1-r\right)\left(W-C_{p}+T_{P}+\beta F\right)\\ +\left(1-x\right)\left(1-y\right)\left(1-r\right)\left(W-C_{p}+\beta F\right)\\ =W-C_{p}+F+yT_{p}-xF,\\ U_{32}=xyr\left(W+T_{P}\right)+x\left(1-y\right)rW\\ +\left(1-x\right)yr\left(W-I_{p}+T_{P}\right)\\ +\left(1-x\right)\left(1-y\right)r\left(W-I_{p}\right)\\ +xy\left(1-r\right)\left(W+T_{P}\right)\\ +x\left(1-y\right)\left(1-r\right)W\\ +\left(1-x\right)y\left(1-r\right)\left(W-\alpha I_{p}+T_{P}\right)\\ +\left(1-x\right)\left(1-y\right)\left(1-r\right)W\\ =W+yT_{p}-\left(1-x\right)rI_{p}-\left(1-x\right)y\left(1-r\right)I_{p},\\ \bar{U_{3}}=zU_{31}+\left(1-z\right)U_{32}. \end{array}$$

According to the Malthusian dynamic equation, the replication dynamic equation of e-commerce platform is obtained as follows:

(10) $$\begin{array}{c} F\left(z\right)=\frac{\text{d}z}{\text{d}t}\\ =z\left(U_{31}-\bar{U_{3}}\right)\\ =z\left(1-z\right)\left\{\beta F-C_{p}+yT_{p}-x\beta F-\left(1-x\right)rI_{p}-\left(1-x\right)y\left(1-r\right)I_{p}\right\}. \end{array}$$

The first partial derivative of *F* (*z*) for *z* is as follows:

(11) $$\begin{array}{c} F_{z}^{\prime }\left(z\right)=\left(1-2z\right)\left\{\beta F-C_{p}+yT_{p}-x\beta F-\left(1-x\right)rI_{p}-\left(1-x\right)y\left(1-r\right)I_{p}\right\}. \end{array}$$

Based on the stability theorem of differential equations, e-commerce platform implements the strategy of information supervision in the stable state must meet the conditions: *F*(*z*) = 0, and *F*_*z*_′(z) <0.

**Proposition 3 .**

When *y* *>* *y*_*0*_, the e-commerce platform will choose information supervision as the stable strategy; when *y* *<* *y*_*0*_, the e-commerce platform will choose information nonsupervision as the stable strategy; when *y* *=* *y*_*0*_, the e-commerce platform cannot determine the stable strategy. Where the threshold is as follows:

(12) $$\begin{array}{c} y_{0}=\frac{C_{p}+x\beta F+\left(1-x\right)rI_{p}-\beta F}{T_{p}-\left(1-x\right)\left(1-r\right)I_{p}}. \end{array}$$

**Proof**

Assume *H*(*y*)=*F* − *C*_*p*_+*yT*_*p*_ − *xF* − (1 − *x*)*rI*_*p*_ − (1 − *x*)*y*(1 − *r*)*I*_*p*_, when *T*_*p*_ − (1 − *x*)(1 − *r*)*I*_*p*_ > 0, (∂*H*/∂*x*) >0, *H (y)* is considered to be an increasing function of *y*. When *y* *>* *y*_*0*_, *H (y)* *>* 0, *F*(*z*)*|*_*z*=1_=0, and *F*_*z*_′(*z*)*|*_*z*=1_ < 0, so *z* = 1 has stability; When *y* *<* *y*_*0*_, *H (y)* < 0, *F*(*z*)*|*_*z*=0_=0, and *F*_*z*_′(*z*)*|*_*z*=0_ < 0, so *z* = 0 has stability; When *z* *=* *z*_*0*_, *H (y)* = 0, *F*(*z*)=0, and *F*_*z*_′(*z*)=0, so *z* is stable at all levels in the range of 0 to 1, that is, the e-commerce platform's strategy does not change over time, regardless of the proportion of e-commerce platform choosing information supervision.

Proposition 3 states that the increase of the proportion of consumer loyalty will change the stable strategy of e-commerce platform from information nonsupervision to information supervision. Similarly, the decline of the proportion of consumer loyalty will change the stable strategy of e-commerce platform from information supervision to information nonsupervision. Therefore, if the consumer can be loyal to the e-commerce company in the platform, the platform will also actively supervise its subordinate company.

**Figure 4:**
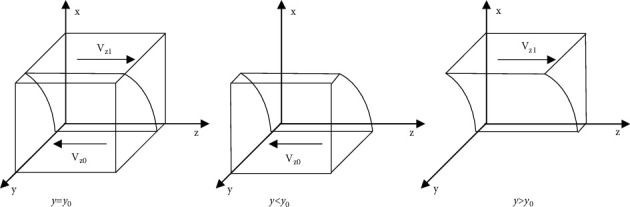
Phase diagram of strategy evolution of e-commerce platform.

Based on Proposition 3, the phase diagram of the strategy evolution of the e-commerce platform is shown in Figure 4.

Inference 3: with the increase of the value of *F*, *β*, and *T*_*p*_, the e-commerce platform is more inclined to implement the information supervision strategy, when other parameters remain unchanged. Similarly, with the increase of the value of *C*_*p*_ and *I*_*p*_, the e-commerce platform is more inclined to implement the information nonsupervision strategy. It shows that the proportion of e-commerce platform implementing information supervision strategy is directly proportional to the fines imposed by the platform for differential pricing of e-commerce company, the proportion of fines imposed by the e-commerce platform for differential pricing of e-commerce company, and the reputation value brought by the loyal consumer to the platform, and inversely proportional to the cost of the platform's information supervision on e-commerce company and the fines by government imposed on the platform for nonsupervision of e-commerce company information resulting in differential pricing.

**Proof**

Since *y*_0_=(*C*_*p*_+(1 − *x*)*rI*_*p*_ − (1 − *x*)*βF*/*T*_*p*_ − (1 − x)(1 − r)I_p_), the volume of *V*_*z1*_ in Figure 4 represents the proportion of information supervision of e-commerce company by the platform, and the corresponding volume of *V*_*z0*_ represents the proportion of information nonsupervision by the platform. When the value of *F*, *β*, and *T*_*p*_ gradually increase, the value of *y*_*0*_ will gradually decrease, and the volume of *V*_*z1*_ will increase at this time, indicating that the proportion of e-commerce platform to implement information supervision increases; When the value of *C*_*p*_ and *I*_*p*_ gradually increases, the value of *y*_*0*_ will gradually increase, and the volume of *V*_*z1*_ will decrease at this time, indicating that the proportion of e-commerce platform to implement information supervision decreases.

#### 3.4.4. Strategy Stability Analysis of Government Regulatory Department

Assuming that the expected benefit of government regulatory department when government implementing the strategy of strictly supervising is *U*_*41*_, the expected benefit of government regulatory department when government implementing the strategy of loosely supervising is *U*_*42*_, and the average expected benefit of the government regulatory department is $\bar{U_{4}}$, which are defined as follows:

(13) $$\begin{array}{c} U_{41}=xyz\left(S-C_{g}+R\right)+x\left(1-y\right)z\left(S-C_{g}+R\right)\\ +\left(1-x\right)yz\left(S-C_{g}+I_{e}\right)+\left(1-x\right)\left(1-y\right)z\left(S-C_{g}+I_{e}\right)\\ +xy\left(1-z\right)\left(S-C_{g}+R\right)+x\left(1-y\right)\left(1-z\right)\left(S-C_{g}+R\right)\\ +\left(1-x\right)y\left(1-z\right)\left(S-C_{g}+I_{e}+I_{p}\right)\\ +\left(1-x\right)\left(1-y\right)\left(1-z\right)\left(S-C_{g}+I_{e}+I_{p}\right)\\ =S-C_{g}+xR+\left(1-x\right)I_{e}+\left(1-x\right)\left(1-z\right)I_{p}.\\ U_{42}=xyzS+x\left(1-y\right)zS+\left(1-x\right)yz\left(S-N+\alpha I_{e}\right)\\ +\left(1-x\right)\left(1-y\right)z\left(S-N\right)+xy\left(1-z\right)S\\ +x\left(1-y\right)\left(1-z\right)S+\left(1-x\right)y\left(1-z\right)\left(S-N+\alpha I_{e}+\alpha I_{p}\right)\\ +\left(1-x\right)\left(1-y\right)\left(1-z\right)\left(S-N\right)\\ =S-\left(1-x\right)N+\left(1-x\right)y\alpha \left[I_{e}+\left(1-z\right)I_{p}\right],\\ \bar{U_{4}}=rU_{41}+\left(1-r\right)U_{42}. \end{array}$$

According to the Malthusian dynamic equation, the replication dynamic equation of the government regulatory department is obtained as follows:

(14) $$\begin{array}{c} F\left(r\right)=\frac{\text{d}r}{\text{d}t}\\ =r\left(U_{41}-\bar{U_{4}}\right)\\ =r\left(1-r\right)\left\{-C_{g}+xR+\left(1-x\right)I_{e}+\left(1-x\right)\left(1-z\right)I_{p}+\left(1-x\right)N-\left(1-x\right)y\alpha \left[I_{e}+\left(1-z\right)I_{p}\right]\right\}. \end{array}$$

The first partial derivative of *F (r)* for *r* is as follows:

(15) $$\begin{array}{c} F_{r}^{\prime }\left(r\right)=\left(1-2r\right)\left\{-C_{g}+xR+\left(1-x\right)I_{e}+\left(1-x\right)\left(1-z\right)I_{p}+\left(1-x\right)N-\left(1-x\right)y\alpha \left[I_{e}+\left(1-z\right)I_{p}\right]\right\}. \end{array}$$

Based on the stability theorem of differential equations, government regulatory department implements the strategy of strictly supervising in the stable state must meet the conditions: *F*(*r*) = 0, and *F*_*r*_′(*r*) < 0.

**Proposition 4 .**

When *z* *>* *z*_*0*_, the government regulatory department will choose strict supervision as the stable strategy; when *z* *<* *z*_*0*_, the stable strategy of the government regulatory department will choose loose supervision as the stable strategy; when *z* *=* *z*_*0*_, the government regulatory department cannot determine the stable strategy. Where the threshold is as follows:

(16) $$\begin{array}{c} z_{0}=\frac{-C_{g}+xR+\left(1-x\right)\left(1-\alpha y\right)I_{p}+\left(1-x\right)\left[\left(1-\alpha y\right)I_{e}+N\right]}{\left(1-x\right)\left(1-\alpha y\right)I_{p}}. \end{array}$$

**Proof**

Assume *H*(*z*)=−*C*_*g*_+*xR*+(1 − *x*)*I*_*e*_+(1 − *x*)(1 − *z*)*I*_*p*_+(1 − *x*)*N* − (1 − *x*)*yα*[*I*_*e*_+(1 − *z*)*I*_*p*_], when (∂*H*/∂*x*) < 0, *H* (*z*) is considered to be an increasing function of *z*. When *z* *<* *z*_*0*_, *H (z)* *>* 0, *F*(*r*)*|*_*r*=1_=0, and *F*_*r*_′(*r*)*|*_*r*=1_ < 0, so *r* = 1 has stability; When *z* *>* *z*_*0*_, *H (z)* < 0, *F*(*r*)*|*_*r*=0_=0, and *F*_*r*_′(*r*)*|*_*r*=0_ < 0, so *r* = 0 has stability; When *z* *=* *z*_*0*_, *H (z)* = 0, *F*(*r*)=0, and *F*_*r*_′(*r*)=0, so *z* is stable at all levels in the range of 0 to 1, that is, the government regulatory department's strategy does not change over time, regardless of the proportion of government regulatory department choosing to strict supervision.

Proposition 4 states that the decline of the proportion of information supervision of e-commerce company by e-commerce platform will change the stable strategy of government regulatory department from loose supervision to strict supervision; Similarly, the increase of the proportion of information supervision of e-commerce company by e-commerce platform will change the stable strategy of government regulatory department from strictly supervising to loosely supervising. Therefore, the government's strict supervision on e-commerce company is the necessary measure under the unfavorable conditions of the e-commerce platform's information supervision on e-commerce company.

**Figure 5:**
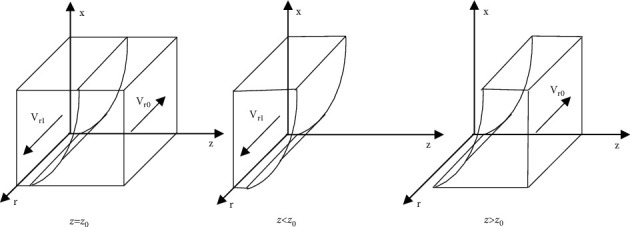
Phase diagram of strategy evolution of government regulatory department.

Based on Proposition 4, the phase diagram of strategy evolution of the government regulatory department is shown in Figure 5.

Inference 4: With the increase of the value of *R*, *I*_*e*_, *I*_*p*_, and *N*, the government regulatory department is more inclined to implement the strict supervision strategy, when other parameters remain unchanged. Similarly, with the increase of the value of *C*_*g*_ and *α*, the government is more inclined to implement the loose supervision strategy. It shows that the proportion of government regulatory department implementing strict supervision strategy is directly proportional to the social benefits obtained, the fines punished by the government on e-commerce company and platform, and the social reputation loss caused by differential pricing under the government's loose supervision, and inversely proportional to the cost for the government to strictly supervise and the proportion of consumer discovering differential pricing.

**Proof**

Since *z*_0_=1 − (*C*_*g*_ − *xR* − (1 − *x*)[(1 − *αy*)*I*_*e*_+*N*]/(1 − *x*)(1 − *αy*)*I*_*p*_), the volume of *V*_*r1*_ in Figure 5 represents the proportion of strictly supervised by the government, and the corresponding volume of *V*_*r0*_ represents the proportion of loosely supervised by government. When the value of *R*, *I*_*e*_, *I*_*p*_, and N gradually increases, the value of *z*_*0*_ will gradually increase, and the volume of *V*_*r1*_ will increase at this time, indicating that the proportion of strict supervision by government regulatory department increases; When the value of *C*_*g*_ and *α* gradually increase, the value of *z*_*0*_ will gradually decrease, and the volume of *V*_*r1*_ will decrease at this time, indicating that the proportion of strict supervision by government regulatory department increases decreases.

## 4. Results and Discussion

### 4.1. ESS Analysis among Four-Party Game Players

In the dynamic system of government regulatory department, e-commerce platform, e-commerce company and consumer, the stability of the strategic combination of the four-party game subjects can be referred to as the nonlinear function stability discriminant method of First Law of Lyapunov. Ritzberger and Weibull [38] and Selten [39] pointed out that the stable solutions in the multi-group evolutionary game are strict Nash equilibrium, which must be the pure strategy. Therefore, this study analyzes 16 pure strategies in four-party evolutionary game learning from the research method of Sun and Su [40].

Due to the replication dynamic equation of each game subject, the Jacobian matrix is obtained as follows:

(17) $$\begin{array}{c} \text{J}=\left[\begin{array}{cccc} F_{x}^{\prime }\left(x\right) & F_{y}^{\prime }\left(x\right) & F_{z}^{\prime }\left(x\right) & F_{r}^{\prime }\left(x\right)\\ F_{x}^{\prime }\left(y\right) & F_{y}^{\prime }\left(y\right) & F_{z}^{\prime }\left(y\right) & F_{r}^{\prime }\left(y\right)\\ F_{x}^{\prime }\left(z\right) & F_{y}^{\prime }\left(z\right) & F_{z}^{\prime }\left(z\right) & F_{r}^{\prime }\left(z\right)\\ F_{x}^{\prime }\left(r\right) & F_{y}^{\prime }\left(r\right) & F_{z}^{\prime }\left(r\right) & F_{r}^{\prime }\left(r\right) \end{array}\right], \end{array}$$

where the elements in the matrix are shown in Appendix A.

#### 4.1.1. ESS Analysis among Four-Party Game Players under the Strict Supervision of Government Regulatory Department

When *C*_*g*_ − *xR* − (1 − *x*)*I*_*e*_ − (1 − *x*)(1 − *z*)*I*_*p*_ − (1 − *x*)*N*+(1 − *x*)*yα*[*I*_*e*_+(1 − *z*)*I*_*p*_] < 0, government regulatory department implements strict supervision. According to the Jacobian matrix shown in Appendix B, the equilibrium solution of the four-party evolutionary game can be obtained, and the stability analysis is shown in Table 3.

  Condition (a): −*P*_*n*_+*P*_*d*_+Δ*P* − *M* − *I*_*e*_*<0*, *C*_*g*_ − *R<0*, and −*C*_*p*_+*T*_*p*_*<*0
  Condition (b): −*P*_*n*_+*P*_*d*_+Δ*P* − *M* − *I*_*e*_ − *F<0*, *C*_*g*_ − *R<0*, and *C*_*p*_ − *T*_*p*_*<*0

It can be seen from Table 3 that there are two possible stable strategies under strict supervision by the government regulatory department, i.e. E5 (1, 1, 0, 1) and E8 (1, 1, 1, 1).

When the condition (a) is met, that is, −*P*_*n*_+*P*_*d*_+Δ*P* − *M* − *I*_*e*_ *<* 0, *C*_*g*_ − *R* *<* 0, and −*C*_*p*_+*T*_*p*_ *<* 0. The sum of the benefits of differential pricing to loyal consumers by e-commerce company is less than the sum of the benefits of nondifferential pricing by e-commerce company to the consumer and the fines to the e-commerce company for differential pricing and compensation of e-commerce company to consumer by the government. The strict supervision cost is less than the social benefits when controlling differential pricing for the government. And the reputation value produced by the loyal consumer to the platform is less than the cost of the platform information supervision. Then the strategy of each subject is stable at equilibrium point E5 (1, 1, 0, 1). E-commerce company implements nondifferential pricing, the consumer is loyal to the e-commerce company, e-commerce platform implements information nonsupervision, and the government strictly supervises e-commerce platform and e-commerce company. This situation may exist in the period of chaotic pricing for the e-commerce company. Since the e-commerce platform benefits less from the information supervision of e-commerce company, it has no motivation to supervise e-commerce company. Therefore, the government must come forward to supervise differential pricing, safeguard consumer rights and interests, and help e-commerce company gain consumer loyalty.

When the condition (b) is met, that is −*P*_*n*_+*P*_*d*_+Δ*P* − *M* − *I*_*e*_ − *F* *<* 0, *C*_*g*_ − *R* *<* 0, and *C*_*p*_ − *T*_*p*_ *<* 0. With the improvement of consumers' awareness of differential pricing and the reduction of the cost of platform supervising information, the information supervision cost of the platform is less than the reputation value brought by the loyal consumer to the platform, and the e-commerce platform can also join into the supervision of e-commerce company. When the other conditions remain unchanged, the strategy of each subject is stable at equilibrium point E8 (1, 1, 1, 1). The government and e-commerce platform jointly strengthen the supervision of differential pricing of e-commerce company, so that e-commerce company inclined to to be nondifferential pricing, and consumer is loyal to the e-commerce company.

#### 4.1.2. ESS Analysis among Four-Party Game Players under the Loose Supervision of Government Regulatory Department

When *C*_*g*_ − *xR* − (1 − *x*)*I*_*e*_ − (1 − *x*)(1 − *z*)*I*_*p*_ − (1 − *x*)*N*+(1 − *x*)*yα*[*I*_*e*_+(1 − *z*)*I*_*p*_] > 0, government regulatory department implements loosely supervision. According to the Jacobian matrix shown in Appendix C, the equilibrium solution of the four-party evolutionary game can be obtained, and the stability analysis is shown in Table 4.

Condition (c): −*P*_*n*_+*P*_*d*_+Δ*P* − *α*(*M*+*I*_*e*_) − *F* <0, *C*_*p*_ − *T*_*p*_ <0, and −*C*_*g*_+*R* <0.

As shown in Table 4 that there is a possible stabilization strategy under loose supervision by government regulatory authorities, i.e. E16 (1, 1, 1, 0).

When the condition (c) is met, that is, −*P*_*n*_+*P*_*d*_+Δ*P* − *α*(*M*+*I*_*e*_) − *F<*0, *C*_*p*_ − *T*_*p*_*<*0, and −*C*_*g*_+*R* <0. The sum of the benefits of e-commerce company's differential pricing for the loyal consumer is less than the sum of the benefits of e-commerce company's nondifferential pricing for consumer, the fines punished by government regulatory department under loosely supervising and the compensation for the consumer for differential pricing of e-commerce company, and the fines imposed by e-commerce platform on the e-commerce company. The reputation value brought by the loyal consumer to the platform is greater than the cost of the platform information supervision. And the strict supervision cost is greater than the social benefits when controlling differential pricing for the government. Then the strategy of each subject is stable at equilibrium point E16 (1, 1, 1, 0). This situation may exist in the normative period of discriminatory pricing by the e-commerce company. At this time, as the proportion of the differential pricing of e-commerce company gradually decreases, the social benefits of the government's strict supervision of differential pricing decrease. When the social benefit is less than the strictly supervising cost of the government regulatory department, the strategy of the government regulatory department will change from strictly supervising to loosely supervising. The main responsibility of supervision will be transferred from the government to the e-commerce platform and consumer. Supervision and fines by e-commerce platform and consumer enable e-commerce company to conduct nondifferential pricing and promote the virtuous circle of the e-commerce industry ecosystem.

### 4.2. Numerical Simulation Analysis

In order to test the reliability of the model and more intuitively demonstrate the influence of key factors in the replication dynamic system on the evolutionary trajectory of stakeholders of the multi-party game, the model is given numerical value combined with the actual situation, and the numerical simulation is carried out by MATLAB2021.

For the e-commerce company operating in the e-commerce platform, the benefit of nondifferential pricing to the consumer is set as *P*_*n*_ = 10, and the benefit of differential pricing to the consumer is set as *P*_*d*_ = 9, and the additional benefit of differential pricing to the loyal consumer is set as *∆P* = 5. If differential pricing is discovered by the government, the compensation of the e-commerce company to the consumer is set as *M* = 4. The reputation value brought by the loyal consumer to the e-commerce company is set as *T*_*e*_ = 5, and the reputation value brought by the loyal consumer to the e-commerce platform is set as *T*_*p*_ = 5. The utility obtained by the loyal consumer when purchasing goods from the e-commerce company is set as *U*_*l*_ = 12, and the utility obtained by the disloyal consumer when purchasing goods from the e-commerce company is set as *U*_*d*_ = 11. The probability of loyal consumer discovering differential pricing under government loose supervision is set as *α* = 0.2 and the complaint cost of the loyal consumer is set as *C*_*c*_ = 3. The social benefit of nondifferential pricing obtained by the government under strict supervision is set as *R* = 7, and the cost of strictly supervised by the government is set as *C*_*g*_ = 6. The fine by government regulatory department for differential pricing of e-commerce company *I*_*e*_ = 3. The social reputation loss of the government caused by differential pricing under loose supervision is set as *N* = 8. The normal benefit obtained by the government from the operation of the platform is set as *S* = 6. The benefit of the platform reasonably providing information to e-commerce company is set as *W* = 5, and the cost of the platform's information supervision on e-commerce company is set as *C*_*p*_ = 7. The fine imposed by the platform to e-commerce company for differential pricing during information supervision is set as *F* = 3, and the proportion of fine imposed by the e-commerce platform for differential pricing of the e-commerce company is set as *β* = 0.6.

#### 4.2.1. The Influence of Government Supervision Mechanism

**Figure 6:**
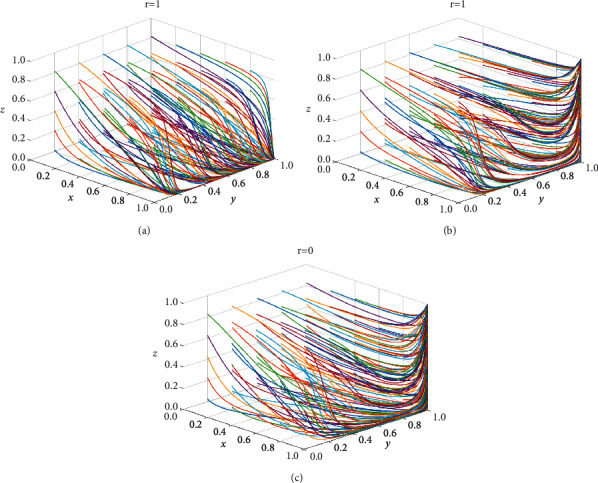
Influence of the establishment of government supervision mechanism on strategy evolution of all parties.

To test whether the government supervision mechanism is effective in the process of differential pricing of e-commerce company, the proportions of government strict supervision are set as *r* = 0 and *r* = 1 to represent the two states of loose supervision and strict supervision of government supervision department. The evolution process of different initial strategies of the e-commercial company, consumer, and e-commerce platform is simulated and analyzed in three-dimensional space, and the simulation results with time are shown in Figure 6.

As shown in Figure 6(a) that when government regulatory department adopts the strict supervision strategy on the differential pricing of e-commerce company, although the e-commerce platform does not take information supervision strategy on account of the high cost for information supervision, the strategies of the e-commerce company and consumer can still incline to be stable in nondifferential pricing and loyalty. This shows that it is very necessary and effective for the government to adopt the strict supervision strategy. With the reduction of *C*_*p*_, that is, the information supervision cost reduced, the platform will be inclined to adopt the strategy of information supervision, to achieve coordinated supervision to e-commerce company by the government and platform, then the company adopts nondifferential pricing, and consumer is loyal to the e-commerce company. And the stable strategy portfolio is demonstrated in Figure 6(b). As is exhibited in Figure 6(c) that when government regulatory department implements the loosely supervising to e-commerce company for the differential pricing due to the high cost of strict supervision, if *C*_*p*_ is small, that is, the cost of information supervision on the e-commerce platform is small, and *α* is at a high level, the consumer can actively discover the differential pricing of the e-commerce company and report it, the e-commerce company will also incline to nondifferential pricing. Therefore, although the government selects the loose supervision strategy, the differential pricing behavior of e-commerce company is supervised collaboratively by the platform and consumer. The strategy equilibrium is consistent with the previous analysis of the stability under different government supervision strategies.

#### 4.2.2. The Influence of Information Supervision Cost of E-Commerce Platforms

**Figure 7:**
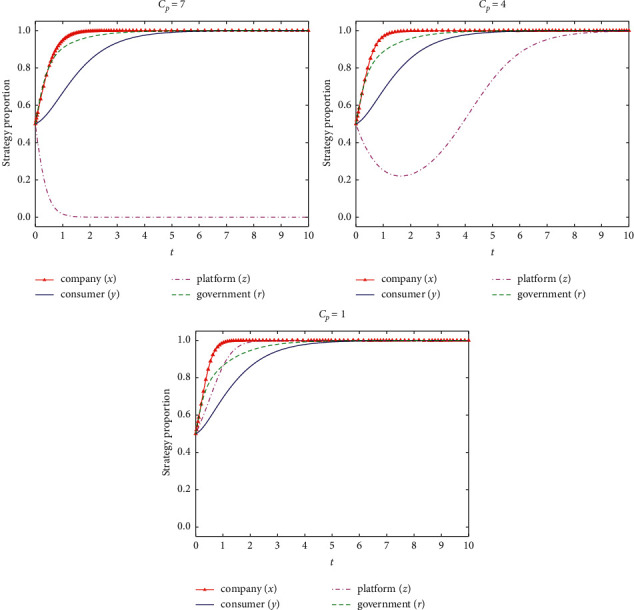
Influence of information supervision cost of e-commerce platform on strategy evolution of all parties.

If *C*_*p*_ = {7, 4, 1}, the stability of the system evolution of the four-party game subjects and the simulation results are shown in Figure 7.

According to Figure 7, with the reduction of the information supervision cost of the e-commerce platform, the supervision strategy of the platform will be transformed from information nonsupervision on e-commerce company to information supervision. Therefore, the platform can join the ranks of the government to regulate the company, and collaboratively supervise the differential pricing of the e-commerce company for loyal consumer. Moreover, the less the information supervision cost of the platform, the faster the stable strategy of information supervision. Therefore, active measures can be adopted to lower the cost for information supervising of e-commerce platform, to stimulate e-commerce platform to supervise the differential pricing behavior of e-commerce company on the platform.

#### 4.2.3. The Influence of the Strict Supervision Cost of Government Regulatory Department

**Figure 8:**
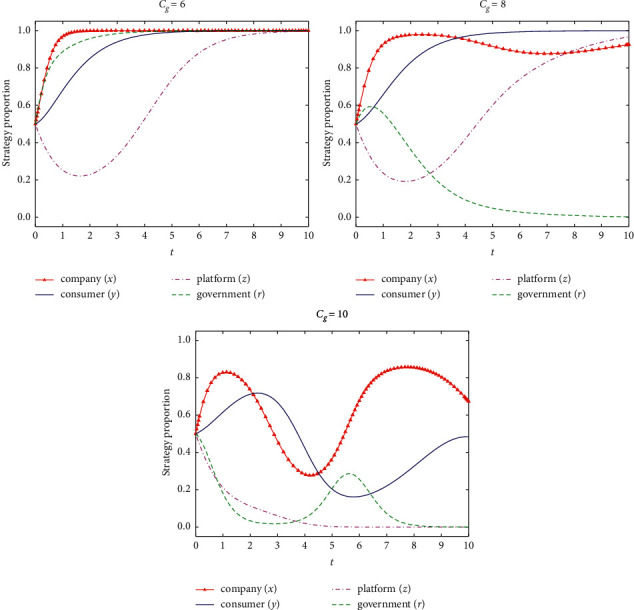
Influence of strict supervision cost of government regulatory department on strategy evolution of all parties.

If *C*_*g*_ = {6, 8, 10}, the stability of the system evolution of the four-party game subjects and the simulation results are shown in Figure 8.

According to Figure 8, the strict supervision cost of government affects the decision-making of government regulatory department, as well as affects the evolution of decision-making of the other subjects. With the increase of government supervision cost, the supervision strategy of the government regulatory department to the differential pricing of e-commerce company will be transformed from strict supervision to loose supervision, and gradually become the cyclical alternating strategy between strict supervision and loose supervision with medium proportion. The strategy of the e-commerce platform will be also transformed from information supervision to information nonsupervision of e-commerce company when strictly supervising cost of government Increasing. Free from the supervision of government regulatory department and platform, the pricing strategy of the company for the loyal consumer will be transformed from nondifferential pricing to moderate-proportion differential pricing, and the strategy change periodically. With the increase of the strictly supervising cost of government, the strategy of the consumer will be transformed from loyalty to e-commerce company to disloyalty. Therefore, the strict supervision cost of the government regulatory department is the key factor in restricting the differential pricing of the e-commerce company. Measures should be arranged to actively reduce the strictly supervising cost of the government regulatory department at a certain level, to stimulate platform and the consumer to regulate the behavior of e-commerce company in differential pricing.

#### 4.2.4. The Influence of the Probability of Loyal Consumer Discovering Differential Pricing under Government's Loose Supervision

**Figure 9:**
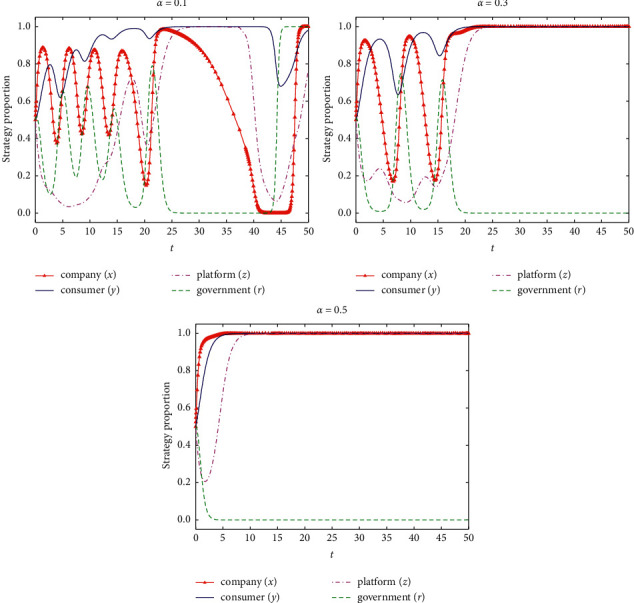
Influence of the probability of loyal consumer discovering differential pricing under government loose supervision on strategy evolution of all parties.

If *α* = {0.1, 0.3, 0.5}, the stability of the system evolution of the four-party game subjects and the simulation results are shown in Figure 9.

According to Figure 9, with the increase of probability of loyal consumer discovering differential pricing under government loose supervision, the probability of exposure of differential pricing behavior of e-commerce company for loyal consumer increases, which will make e-commerce company gradually improve the proportion of nondifferential pricing and stabilize in the nondifferential pricing strategy. The e-commerce platform can also gradually improve the proportion of information supervision due to the increase of fines for nonsupervision of e-commerce company information resulting in differential pricing, and the behavior stabilizes in the information supervision strategy. The government regulatory department can gradually loose supervision and transfer the responsibility of supervision to e-commerce platform and the consumer. Therefore, measures can be taken to encourage the consumer to report the differential pricing behavior of e-commerce company, to maintain the stable and sustainable progress of e-commerce platform and systems.

#### 4.2.5. The Influence of the Penalties for Differential Pricing of E-Commerce Company under Government's Loose Supervision

**Figure 10:**
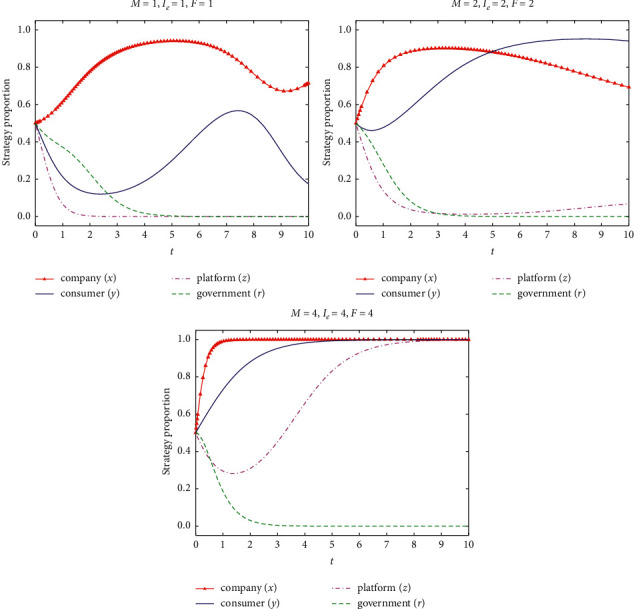
Influence of the penalties for differential pricing of e-commerce company under government loose supervision on strategy evolution of all parties.

If *M* = {1, 2, 4}, *I*_*e*_ = {1, 2, 4}, and *F* = {1, 2, 4}, the evolution process and results of the strategy of the four-party game subjects are shown in Figure 10.

According to Figure 10, with the increase of the fines given by consumer, e-commerce platform, and government regulatory department for differential pricing of e-commerce company, the e-commerce company will gradually increase the proportion of nondifferential pricing and stabilize in the nondifferential pricing strategy. The consumer will increase the proportion of loyalty to the e-commerce company and the behavior stabilize in the loyalty strategy when the compensation for differential pricing from e-commerce company increases to compensate for the loss of differential pricing. The e-commerce platform will also gradually improve the proportion of information supervision due to the increase of benefits from information supervision fines and the behavior stabilizes in the information supervision strategy. Therefore, the nondifferential pricing behavior of e-commerce company can be promoted by increasing the punishment for differential pricing, to realize the joint dynamic supervision of the e-commerce platform, the consumer, and the government on the pricing of the e-commerce company.

## 5. Conclusions

Given the phenomenon of “big data killing” that e-commerce companies use customer information in the pricing process, this paper studies how to safeguard consumers' pricing fairness in the context of the Internet, and builds the four-party evolutionary game model for the supervision on differential pricing of e-commerce company, analyzes the stability of the strategy selection of each subject in the model, and the stability of equilibrium point of the strategic combination in the replication dynamic system, and simulates and analyzes the influence of key elements on the strategy evolution. The main conclusions are as follows:

1. The government supervision mechanism can play an effective role to limit differential pricing of the e-commerce company. When the proportion of strict government supervision adds, the sum of the benefits of differential pricing for loyal consumers by e-commerce company is less than the penalty cost of e-commerce company, and strict supervision cost of government is less than its social benefits, then e-commerce company inclines more to choose the strategy of nondifferential pricing. Since the reputation value of the e-commerce platform is less than the information supervision cost of platform, the platform inclines more to conduct information nonsupervision. Therefore, the equilibrium strategy of each subject is stable at point E5 (1, 1, 0, 1), which occurs in the early stage of the government's strict supervision on the e-commerce company. With the reduction of the supervision cost of the platform, it is also willing to join the supervision on differential pricing of e-commerce company for the platform and inclines more to choose information supervision strategy. Therefore, the equilibrium strategy of each subject is stable at point E8 (1, 1, 1, 1), which occurs in the stable stage of the government's strict supervision of e-commerce company, and the participation of the e-commerce platform relieved the pressure on government supervising on the company. When the strictly supervising cost of government increases, the reputation value of the platform is greater than the supervision cost of the platform, then the government regulatory department inclines more to loose supervision strategy. Therefore, the equilibrium strategy of each subject is stable at point E16 (1, 1, 1, 0), which occurs in the later stage of the government's strict supervision of e-commerce company. When both e-commerce platform and consumer realize the important role of supervision and conduct strong collaborative supervision, the government can take the way of auxiliary supervision to control the differential pricing of the e-commerce company.
2. The information supervision cost of the e-commerce platform is the main factor affecting the supervision strategy of the platform. When the supervision cost of the platform is greater than the reputation value of the platform, the platform inclines more to conduct information nonsupervision. However, as the information supervision cost of the platform decreases and is less than the reputation value of the platform, the stable strategy of the e-commerce platform will transform into information supervision and then promote nondifferential pricing for the e-commerce company. Moreover, the less the information supervision cost of the e-commerce platform, the faster the stable strategy of the e-commerce platform can transform into the information supervision strategy.
3. The strict supervision cost of government is the main factor affecting the strategies of all parties. When the strict supervision cost of government is so small as to be less than the social benefits of government strict supervision on differential pricing of e-commerce company, the equilibrium strategy of all parties is that both government regulatory department and e-commerce platform implement supervision, e-commerce company conduct nondifferential pricing, and consumer is loyal to the e-commerce company. However, when the strict supervision cost of government increases and exceeds the social benefits of government strict supervision on differential pricing of e-commerce company, the government gradually inclines to loose supervision strategy. At this moment, if platform and consumer can supervise the pricing of the e-commerce company to a certain extent, e-commerce company still incline to nondifferential pricing strategy. When the strict supervision cost of government increases to a very high level, not only the government cannot strictly supervise, but also e-commerce platform will not supervise the information used by the e-commerce company. Then e-commerce company will incline to differentiate pricing, and the consumer will be disloyal.
4. The probability of the consumer discovering differential pricing under the government's loose supervision policy is an important factor affecting the strategies of all parties. As strict supervision cost of government is at a higher level, and the probability of consumer discovering differential pricing of the e-commerce company is small, neither the government nor the e-commerce platform can incline to the more stable behavioral strategy. Although customer inclines to be loyal to the e-commerce company, the strategies of four subjects cannot maintain the stable equilibrium, and the strategy of e-commerce company become cyclical alternating between differential pricing and nondifferential pricing. When the probability of consumer discovering differential pricing of e-commerce company increases, e-commerce company gradually inclines to nondifferential pricing strategy, e-commerce platform gradually inclines to information supervision strategy, and the government gradually inclines to loose supervision strategy. The equilibrium strategy of four-party behavior achieves. The higher the probability level of consumer discovering differential pricing, the faster the equilibrium strategy of four-party behavior achieves. This conclusion also confirms the conclusion in the research of Yu and Li [9] and Wu et al. [30] that the probability of consumer finding himself killed in price is the important factor affecting the strategy choice of consumer and company.
5. The penalties for differential pricing of e-commerce company under the government's loose regulatory are the important factors affecting the strategies of all parties. When consumers, e-commerce platform and government regulatory department impose the fines and compensation on differential pricing of e-commerce companies at a low level, the government and e-commerce platform incline to not supervise, and e-commerce company and consumer cannot maintain a stable equilibrium. When the penalties for differential pricing of e-commerce company is high, e-commerce platform inclines to supervise the information, consumer inclines to be loyal, while e-commerce company inclines to price nondifferentially, the government inclines to loose supervise, and the strategies remains stable. The higher the penalties for differential pricing of e-commerce company, the faster the equilibrium strategy of four-party behavior achieves.

In this study, the modeling analysis and simulation of the supervision of “big data killing” of e-commerce company are carried out, which breaks through the limitation of analyzing only two or three parties in the existing “big data killing” problem. It is a beneficial supplement to systematic research on this issue that more participants consider their action strategies under the same system. The four-party evolutionary game model constructed also expands the application scope of the evolutionary game method in the study of pricing supervision of e-commerce company. The research conclusions can provide favorable theoretical support for the “big data killing” problem in practice.

Therefore, to better restrain the pricing behavior of e-commerce company, regulate differential pricing, and build a good e-commerce shopping environment, the following measures should be taken by the government regulatory department, e-commerce platform, e-commerce company, and consumer.

1. From the perspective of the government, the government regulatory department must supervise e-commerce company, especially in the early stage of price discrimination by using customer information. Therefore, the government needs to use economic and policy means to effectively manage the operation of e-commerce platform and company and promote the enthusiasm of e-commerce company to conduct nondifferential pricing. For example, adopting more advanced big data analysis technology to supervise price changes of e-commerce company; establishing more extensive and efficient reporting channels so that consumers can timely price complaints; improving corresponding legal measures to increase the violation cost of the e-commerce company and punishing “big data killing” from the aspects of economy and reputation. While supervising, it is also necessary to pay attention to reducing the strict supervision cost of the government regulatory department.
2. From the perspective of the e-commerce platform, as the important carrier of e-commerce operation, the e-commerce platform should strengthen information supervising of the e-commerce company. The e-commerce platform is the main body that controls customer information. E-commerce company conducts “big data killing” differential pricing based on the mastery of customer information. Therefore, the e-commerce platform needs to carry out information supervision when providing information for the e-commerce company and formulates policies to punish e-commerce company with differential pricing. It is also necessary to improve the technical management level of the e-commerce platform, and use innovative technology based on big data to monitor e-commerce company and reduce the supervision cost of the e-commerce platform.
3. From the perspective of consumers, they should actively protect their rights and interests. While online shopping brings convenience to consumers, it may also lead to the possibility of price discrimination with consumer information. In the process of e-commerce shopping, consumers will prefer some e-commerce companies due to path dependence, and then form customer loyalty, but this path dependence should not be the reason for the differential pricing of e-commerce companies. Therefore, consumers should enhance price sensitivity and verify the displayed price of e-commerce companies through various channels, to reduce the infringement of consumer rights and interests by e-commerce companies.
4. From the perspective of the e-commerce company, although maximizing profits is the important motive of business behavior, the reputation and service in e-commerce shopping are the foundation for the long-term development of the e-commerce company. Under the market conditions where consumers' transfer costs are getting lower and lower, the e-commerce company can grow gradually mainly based on gaining the loyal customer. Therefore, e-commerce company should not adopt the differential pricing strategy in pursuit of temporary benefits. Although the economic benefits brought by nondifferential pricing of e-commerce company are less in the short term, the reputation benefits and social benefits can create greater economic benefits for the development of the company in the long term, which are the wealth of e-commerce company. The reputation benefits and social benefits brought by nondifferential pricing can be benefit for the more fair and equitable overall development environment for e-commerce.

This study systematically analyzes the model on the supervision of “big data killing” in the e-commerce company. However, the mechanism setting of the four-party game in the study has been simplified to a certain extent, and the strategy space needs to be more detailed and in-depth, which should be improved in the future. Moreover, because the simulation data were conducted under simulated conditions according to actual conditions, there may be some deviations in the effectiveness of players' behavior analysis in the “big data killing” game. In the future, methods such as data mining will be used to collect big data, and empirical analysis of evolutionary game will be carried out, to improve the research on the participants behavior of “big data killing” in e-commerce transactions.

## Figures and Tables

**Table 1:** Parameter description.

Parameter	Description
*P*_*n*_	The benefit of nondifferential pricing by e-commerce company to consumer
*P*_*d*_	The benefit of differential pricing by e-commerce company to consumer
*∆P*	The additional benefit of differential pricing by e-commerce company to the loyal consumer
*M*	Compensation of e-commerce company to the loyal consumer for differential pricing
*T*_*e*_	The reputation value of the loyal consumer to e-commerce company
*U*_*l*_	The utility obtained by the loyal consumer from purchasing goods
*U*_*d*_	The utility obtained by the disloyal consumer from purchasing goods
*C*_*c*_	The cost of consumer complaint
*α*	Probability of loyal consumer discovering differential pricing under government loose supervision, and *α* ∈ [0,1]
*C*_*g*_	The cost of strict supervision by the government regulatory department
*N*	Social reputation loss caused by differential pricing under government loose supervision
*R*	The social benefit of nondifferential pricing under the government strict supervision
*I*_*e*_	Fine by government regulatory department for differential pricing to e-commerce company
*I*_*p*_	Fine by government imposed on the platform for nonsupervision of e-commerce company information resulting in differential pricing
*S*	The normal benefit obtained by the government from the operation of the e-commerce platform
*W*	The benefit of the platform reasonably providing information to the e-commerce company
*C*_*p*_	The cost of the platform's information supervision on the e-commerce company
*F*	Fines imposed by the platform to e-commerce company for differential pricing during information supervision
*β*	The proportion of the fine imposed by the e-commerce platform for differential pricing of e-commerce company, *β* ∈ [0,1]
*T*_*p*_	The reputation value of the loyal consumer to the e-commerce platform

**Table 2:** Game model benefit matrix of government regulatory department, e-commerce platform, e-commerce company, and consumer

Strategy choice		E-commerce company	Government regulatory department			
			Strict supervision, *r*		Loose supervision, 1 − *r*	
			Loyalty *y*	Disloyalty 1 − *y*	Loyalty *y*	Disloyalty 1 − *y*
E-commerce platform	Information supervision *z*	Nondifferential pricing *x*	*P*_*n*_ *+* *T*_*e*_	*P*_*n*_	*P*_*n*_ *+* *T*_*e*_	*P*_*n*_
			*U*_*l*_	*U*_*d*_	*U*_*l*_	*U*_*d*_
			*W − C*_*p*_ *+* *T*_*p*_	*W − C*_*p*_	*W − C*_*p*_ *+* *T*_*p*_	*W* − *C*_*p*_
			*S* − *C*_*g*_*+R*	*S* − *C*_*g*_ + *R*	*S*	*S*
		Differential pricing 1 − *x*	*P*_*d*_ *+* *∆P* *+* *T*_*e*_ − *M* − *I*_*e*_ − *F*	*P*_*d*_ − *I*_*e*_ − *F*	*P*_*d*_ *+* *∆P* *+* *T*_*e*_ − *αM* − *αI*_*e*_ − *F*	*P*_*d*_ − *F*
			*U*_*l*_ * − ∆P* *+* *M* *+* *(1* − *β)F*	*U*_*d*_	*U*_*l*_ − *∆P* − *C*_*c*_ *+* *αM* *+* *(1* − *β)F*	*U*_*d*_
			*W − C*_*p*_ *+* *βF* *+* *T*_*p*_	*W − C*_*p*_ *+* *F*	*W* − *C*_*p*_ *+* *βF* *+* *T*_*p*_	*W − C*_*p*_ *+* *F*
			*S − C*_*g*_ *+* *I*_*e*_	*S − C*_*g*_ *+* *I*_*e*_	*S* *+* *αI*_*e*_* − N*	*S − N*
	Information nonsupervision 1 − *z*	Nondifferential pricing *x*	*P*_*n*_ *+* *T*_*e*_	*P*_*n*_	*P*_*n*_ *+* *T*_*e*_	*P*_*n*_
			*U*_*l*_	*U*_*d*_	*U*_*l*_	*U*_*d*_
			*W* *+* *T*_*p*_	*W*	*W* *+* *T*_*p*_	*W*
			*S − C*_*g*_ *+* *R*	*S − C*_*g*_ *+* *R*	*S*	*S*
		Differential pricing 1 − *x*	*P*_*d*_ *+* *∆P* *+* *T*_*e*_* − M − I*_*e*_	*P*_*d*_ * − I*_*e*_	*P*_*d*_ *+* *∆P* *+* *T*_*e*_* − αM − αI*_*e*_	*P*_*d*_
			*U*_*l*_ * − ∆P* *+* *M*	*U*_*d*_	*U*_*l*_ * − ∆P − C*_*c*_ *+* *αM*	*U*_*d*_
			*W − I*_*p*_ *+* *T*_*p*_	*W − I*_*p*_	*W − αI*_*p*_ *+* *T*_*p*_	*W*
			*S − C*_*g*_ *+* *I*_*e*_ *+* *I*_*p*_	*S − C*_*g*_ *+I*_*e*_ *+* *I*_*p*_	*S+αI*_*e*_ *+αI*_*p*_ * − N*	*S − N*

**Table 3:** Asymptotic stability analysis of equilibrium point of replication dynamic system under the strict supervision of government regulatory department.

Equilibrium point	Eigenvalue symbol	Stability of equilibrium point
E1 (0, 0, 0, 1)	(+, *X*, *X, X*)	Instability point
E2 (1, 0, 0, 1)	(−, +, −, −)	Instability point
E3 (0, 1, 0, 1)	(+, *X*, *X*, −)	Instability point
E4 (0, 0, 1, 1)	(+, *X*, *X*, −)	Instability point
E5 (1, 1, 0, 1)	(−, −, −, −)	ESS in condition (a)
E6 (1, 0, 1, 1)	(−, +, +,−)	Instability point
E7 (0, 1, 1, 1)	(+, *X*, *X*,−)	Instability point
E8 (1, 1, 1, 1)	(−, −, −, −)	ESS in condition (b)

Note: *X* means uncertain of symbol, and ESS means the evolutionarily stable strategy.

**Table 4:** Asymptotic stability analysis of equilibrium point of replication dynamic system under the loose supervision of government regulatory department.

Equilibrium point	Eigenvalue symbol	Stability of equilibrium point
E9 (0, 0, 0, 0)	(+, *X*, *X, X*)	Instability point
E10 (1, 0, 0, 0)	(−, +, *X*, *X*)	Instability point
E11 (0, 1, 0, 0)	(+, *X*, *X*, *X*)	Instability point
E12 (0, 0, 1, 0)	(+, *X*, *X*, *X*)	Instability point
E13 (1, 1, 0, 0)	(*X*,−, *X*, +*V*)	Instability point
E14 (1, 0, 1, 0)	(*X*, +, +, +)	Instability point
E15 (0, 1, 1, 0)	(+, *X*, *X, X*)	Instability point
E16 (1, 1, 1, 0)	(−, −, −, −)	ESS in condition (c)

Note: *X* means uncertain of symbol, and ESS means the evolutionarily stable strategy.

## Data Availability

The data used to support the findings of the study are available from the corresponding author upon request.

## Appendix

### A. Jacobian Matrix of Replicated Dynamical Systems

(A.1)
$$\begin{array}{c} \text{J}=\left[\begin{array}{cccc} F_{x}^{\prime }\left(x\right) & F_{y}^{\prime }\left(x\right) & F_{z}^{\prime }\left(x\right) & F_{r}^{\prime }\left(x\right)\\ F_{x}^{\prime }\left(y\right) & F_{y}^{\prime }\left(y\right) & F_{z}^{\prime }\left(y\right) & F_{r}^{\prime }\left(y\right)\\ F_{x}^{\prime }\left(z\right) & F_{y}^{\prime }\left(z\right) & F_{z}^{\prime }\left(z\right) & F_{r}^{\prime }\left(z\right)\\ F_{x}^{\prime }\left(r\right) & F_{y}^{\prime }\left(r\right) & F_{z}^{\prime }\left(r\right) & F_{r}^{\prime }\left(r\right) \end{array}\right]. \end{array}$$

Among them,

(A.2) $$\begin{array}{c} F_{x}^{\prime }\left(x\right)=\left(1-2x\right)\left\{P_{n}-P_{d}-y\Delta P+y\left(M+I_{e}\right)\left[r+\left(1-r\right)\alpha \right]+\left(1-y\right)rI_{e}+zF\right\},\\ F_{y}^{\prime }\left(x\right)=x\left(1-x\right)\left\{-\Delta P+\left(M+I_{e}\right)\left[r+\left(1-r\right)\alpha \right]-rI_{e}\right\},\\ F_{z}^{\prime }\left(x\right)=x\left(1-x\right)F,\\ F_{r}^{\prime }\left(x\right)=x\left(1-x\right)\left[y\left(M+I_{e}\right)\left(1-\alpha \right)-yI_{e}\right],\\ F_{x}^{\prime }\left(y\right)=y\left(1-y\right)\left\{\Delta P-\left[M+\left(1-r\right)\alpha M\right]+\left(1-r\right)C_{c}-z\left(1-\beta \right)F\right\},\\ F_{y}^{\prime }\left(y\right)=\left(1-2y\right)\left\{U_{l}-U_{d}-\left(1-x\right)\Delta P+\left(1-x\right)\left[M+\left(1-r\right)\alpha M\right]-\left(1-x\right)\left(1-r\right)C_{c}+\left(1-x\right)z\left(1-\beta \right)F\right\},\\ F_{z}^{\prime }\left(y\right)=y\left(1-y\right)\left\{\left(1-x\right)\left(1-\beta \right)F\right\},\\ F_{r}^{\prime }\left(y\right)=y\left(1-y\right)\left\{\left(\left(1-x\right)\left(-\alpha \right)M\right]+\left(1-x\right)C_{c}\right\},\\ F_{x}^{\prime }\left(z\right)=z\left(1-z\right)\left\{-\beta F+rI_{p}+y\left(1-r\right)I_{p}\right\},\\ F_{y}^{\prime }\left(z\right)=z\left(1-z\right)\left\{T_{p}-\left(1-x\right)\left(1-r\right)I_{p}\right\},\\ F_{z}^{\prime }\left(z\right)=\left(1-2z\right)\left\{\beta F-C_{p}+yT_{p}-x\beta F-\left(1-x\right)rI_{p}-\left(1-x\right)y\left(1-r\right)I_{p}\right\},\\ F_{r}^{\prime }\left(z\right)=z\left(1-z\right)\left\{-\left(1-x\right)I_{p}+\left(1-x\right)yI_{p}\right\},\\ F_{x}^{\prime }\left(r\right)=r\left(1-r\right)\left\{R-I_{e}-N-\left(1-z\right)I_{p}+y\alpha \left[I_{e}+\left(1-z\right)I_{p}\right]\right\},\\ F_{y}^{\prime }\left(r\right)=r\left(1-r\right)\left\{-\left(1-x\right)\alpha \left[I_{e}+\left(1-z\right)I_{p}\right]\right\},\\ F_{z}^{\prime }\left(r\right)=r\left(1-r\right)\left\{-\left(1-x\right)I_{p}+\left(1-x\right)y\alpha I_{p}\right\},\\ F_{r}^{\prime }\left(r\right)=\left(1-2r\right)\left\{-C_{g}+xR+\left(1-x\right)I_{e}+\left(1-x\right)\left(1-z\right)I_{p}+\left(1-x\right)N-\left(1-x\right)y\alpha \left[I_{e}+\left(1-z\right)I_{p}\right]\right\}. \end{array}$$

### B. Jacobian Matrix of Equilibrium Points of Reproduced Dynamic Systems under Government Strict Supervision

The Jacobian matrix of the equilibrium point E1 (0, 0, 0, 1) is as follows:

(B.1) $$\begin{array}{c} J_{1}=\left[\begin{array}{cccc} P_{n}-P_{d}+I_{e} & 0 & 0 & 0\\ 0 & U_{l}-U_{d}-\Delta P+M & 0 & 0\\ 0 & 0 & \beta F-C_{p}-I_{p} & 0\\ 0 & 0 & 0 & -I_{e}+C_{g}-N-I_{p} \end{array}\right]. \end{array}$$

The Jacobian matrix of the equilibrium point E2 (1, 0, 0, 1) is as follows:

(B.2) $$\begin{array}{c} J_{2}=\left[\begin{array}{cccc} -P_{n}+P_{d}-I_{e} & 0 & 0 & 0\\ 0 & U_{l}-U_{d} & 0 & 0\\ 0 & 0 & -C_{p} & 0\\ 0 & 0 & 0 & C_{g}-R \end{array}\right]. \end{array}$$

The Jacobian matrix of the equilibrium point E3 (0, 1, 0, 1) is as follows:

(B.3) $$\begin{array}{c} J_{3}=\left[\begin{array}{cccc} P_{n}-P_{d}+\Delta P+M+I_{e} & 0 & 0 & 0\\ 0 & -U_{l}+U_{d}+\Delta P-M & 0 & 0\\ 0 & 0 & \beta F-C_{p}+T_{p}-I_{p} & 0\\ 0 & 0 & 0 & C_{g}-\left(1-\alpha \right)\left(I_{e}+I_{p}\right)-N \end{array}\right]. \end{array}$$

The Jacobian matrix of the equilibrium point E4 (0, 0, 1, 1) is as follows:

(B.4) $$\begin{array}{c} J_{4}=\left[\begin{array}{cccc} P_{n}-P_{d}+I_{e}+F & 0 & 0 & 0\\ 0 & U_{l}-U_{d}-\Delta P+M+\left(1-\beta \right)F & 0 & 0\\ 0 & 0 & -\beta F+C_{p}+I_{p} & 0\\ 0 & 0 & 0 & C_{g}-I_{e}-N \end{array}\right]. \end{array}$$

The Jacobian matrix of the equilibrium point E5 (1, 1, 0, 1) is as follows:

(B.5) $$\begin{array}{c} J_{5}=\left[\begin{array}{cccc} -P_{n}+P_{d}+\Delta P-M-I_{e} & 0 & 0 & 0\\ 0 & -U_{l}+U_{d} & 0 & 0\\ 0 & 0 & -C_{p}+T_{p} & 0\\ 0 & 0 & 0 & C_{g}-R \end{array}\right]. \end{array}$$

The Jacobian matrix of the equilibrium point E6 (1, 0, 1, 1) is as follows:

(B.6) $$\begin{array}{c} J_{6}=\left[\begin{array}{cccc} -P_{n}+P_{d}-I_{e}-F & 0 & 0 & 0\\ 0 & U_{l}-U_{d} & 0 & 0\\ 0 & 0 & C_{p} & 0\\ 0 & 0 & 0 & C_{g}-R \end{array}\right]. \end{array}$$

The Jacobian matrix of the equilibrium point E7 (0, 1, 1, 1) is as follows:

(B.7) $$\begin{array}{c} J_{7}=\left[\begin{array}{cccc} P_{n}-P_{d}-\Delta P+M+I_{e}+F & 0 & 0 & 0\\ 0 & -U_{l}+U_{d}+\Delta P-M-\left(1-\beta \right)F & 0 & 0\\ 0 & 0 & -\beta F+C_{p}-T_{p}+I_{p} & 0\\ 0 & 0 & 0 & C_{g}-\left(1-\alpha \right)I_{e}-N \end{array}\right]. \end{array}$$

The Jacobian matrix of the equilibrium point E8 (1, 1, 1, 1) is as follows:

(B.8) $$\begin{array}{c} J_{8}=\left[\begin{array}{cccc} -P_{n}+P_{d}+\Delta P-M-I_{e}-F & 0 & 0 & 0\\ 0 & -U_{l}+U_{d} & 0 & 0\\ 0 & 0 & C_{p}-T_{p} & 0\\ 0 & 0 & 0 & C_{g}-R \end{array}\right]. \end{array}$$

### C. Jacobian Matrix of Equilibrium Points of Reproduced Dynamic Systems under Government Loose Supervision

The Jacobian matrix of the equilibrium point E9 (0, 0, 0, 0) is as follows:

(C.1) $$\begin{array}{c} J_{9}=\left[\begin{array}{cccc} P_{n}-P_{d} & 0 & 0 & 0\\ 0 & U_{l}-U_{d}-\Delta P+\left(1+\alpha \right)M-C_{c} & 0 & 0\\ 0 & 0 & \beta F-C_{p} & 0\\ 0 & 0 & 0 & C_{g}-I_{e}-I_{p}-N \end{array}\right]. \end{array}$$

The Jacobian matrix of the equilibrium point E10 (1, 0, 0, 0) is as follows:

(C.2) $$\begin{array}{c} J_{10}=\left[\begin{array}{cccc} -P_{n}+P_{d} & 0 & 0 & 0\\ 0 & U_{l}-U_{d} & 0 & 0\\ 0 & 0 & \beta F-C_{p} & 0\\ 0 & 0 & 0 & -C_{g}+I_{e}+I_{p}+N \end{array}\right]. \end{array}$$

The Jacobian matrix of the equilibrium point E11 (0, 1, 0, 0) is as follows:

(C.3) $$\begin{array}{c} J_{11}=\left[\begin{array}{cccc} P_{n}-P_{d}-\Delta P+\alpha \left(M+I_{e}\right) & 0 & 0 & 0\\ 0 & -U_{l}+U_{d}+\Delta P-\left(1+\alpha \right)M+C_{c} & 0 & 0\\ 0 & 0 & \beta F-C_{p}+T_{p}-I_{p} & 0\\ 0 & 0 & 0 & -C_{g}+\left(1-\alpha \right)\left(I_{e}+I_{p}\right)+N \end{array}\right]. \end{array}$$

The Jacobian matrix of the equilibrium point E12 (0, 0, 1, 0) is as follows:

(C.4) $$\begin{array}{c} J_{12}=\left[\begin{array}{cccc} P_{n}-P_{d}+F & 0 & 0 & 0\\ 0 & U_{l}-U_{d}-\Delta P+\left(1+\alpha \right)M-C_{c}+\left(1-\beta \right)F & 0 & 0\\ 0 & 0 & -\beta F+C_{p} & 0\\ 0 & 0 & 0 & -C_{g}+I_{e}+N \end{array}\right]. \end{array}$$

The Jacobian matrix of the equilibrium point E13 (1, 1, 0, 0) is as follows:

(C.5) $$\begin{array}{c} J_{13}=\left[\begin{array}{cccc} -P_{n}+P_{d}+\Delta P-\alpha \left(M+I_{e}\right) & 0 & 0 & 0\\ 0 & -U_{l}+U_{d} & 0 & 0\\ 0 & 0 & -C_{p}+T_{p} & 0\\ 0 & 0 & 0 & -C_{g}+R \end{array}\right]. \end{array}$$

The Jacobian matrix of the equilibrium point E14 (1, 0, 1, 0) is as follows:

(C.6) $$\begin{array}{c} J_{14}=\left[\begin{array}{cccc} -P_{n}+P_{d}-F & 0 & 0 & 0\\ 0 & U_{l}-U_{d} & 0 & 0\\ 0 & 0 & C_{p} & 0\\ 0 & 0 & 0 & -C_{g}+R \end{array}\right]. \end{array}$$

The Jacobian matrix of the equilibrium point E15 (0, 1, 1, 0) is as follows:

(C.7) $$\begin{array}{c} {\text{J}}_{15}=\left[\begin{array}{cccc} P_{n}-P_{d}+\Delta P+\alpha \left(M+I_{e}\right)+F & 0 & 0 & 0\\ 0 & -U_{l}+U_{d}+\Delta P-\left(1+\alpha \right)M+C_{c}-\left(1-\beta \right)F & 0 & 0\\ 0 & 0 & -\beta F+C_{p}-T_{p}+I_{p} & 0\\ 0 & 0 & 0 & -C_{g}+\left(1-\alpha \right)I_{e}+N \end{array}\right]. \end{array}$$

The Jacobian matrix of the equilibrium point E16 (1, 1, 1, 0) is as follows:

(C.8) $$\begin{array}{c} J_{16}=\left[\begin{array}{cccc} -P_{n}+P_{d}+\Delta P-\alpha \left(M+I_{e}\right)-F & 0 & 0 & 0\\ 0 & -U_{l}+U_{d} & 0 & 0\\ 0 & 0 & C_{p}-T_{p} & 0\\ 0 & 0 & 0 & -C_{g}+R \end{array}\right]. \end{array}$$
